# Reduced gray matter in the prefrontal cortex and hippocampus: a potential neuroanatomical feature of insomnia comorbid with anxiety

**DOI:** 10.3389/fpsyt.2026.1861287

**Published:** 2026-07-22

**Authors:** Minghe Xu, Yanjie Zhou, Bei Feng, Kunlin Ni, Xinyu Li, Pan He, Qingzhu Zhao, Yu Fan, Li Xiao, Bing Yu

**Affiliations:** 1 Department of Radiology, Shengjing Hospital of China Medical University, Shenyang, China; 2 The Sleep Medicine Center, Shengjing Hospital of China Medical University, Shenyang, China

**Keywords:** anxiety, comorbidity, gray matter, insomnia, magnetic resonance imaging

## Abstract

**Background:**

Chronic insomnia disorder (CID) frequently comorbid with generalized anxiety disorder (GAD), and accurate identification of this comorbidity remains challenging. Neuroimaging-based gray matter measurements may provide objective features to improve precise identification.

**Objective:**

To investigate the potential value of gray matter features in distinguishing CID comorbid with GAD from CID or GAD alone.

**Methods:**

Voxel-based morphometry (VBM) and surface-based morphometry (SBM) were used to assess gray matter features. Comparisons were conducted among patients with CID (n = 46), GAD (n = 44), CID comorbid with GAD (n = 43), and healthy controls (n = 45).

**Results:**

CID comorbid with GAD was characterized by reduced gray matter volume in the prefrontal cortex and hippocampus. CID was characterized by reduced hippocampal volume and increased gyrification index (GI) in the prefrontal cortex. GAD was characterized by increased regional prefrontal cortex volume. These neuroanatomical features were associated with disease severity.

**Conclusion:**

Distinct gray matter features were observed among CID, GAD, and CID comorbid with GAD. These findings suggest that gray matter features may contribute to the identification of these conditions.

## 1 Introduction

CID and GAD affect a substantial proportion of the global population. Epidemiological studies indicate that the lifetime prevalence of CID in adults is approximately 10% (1), whereas that of GAD reaches up to 14% (2). Despite their high prevalence, accurate diagnosis in clinical practice remains challenging. This is largely due to the lack of objective diagnostic biomarkers (3). This challenge is further compounded by the frequent comorbidity of CID and GAD (4). In routine clinical settings, patients are often treated with both hypnotics and anxiolytics (5). However, CID and GAD are fundamentally distinct disorders with different underlying pathophysiological mechanisms. CID is primarily associated with dysregulation of sleep-related neural systems, involving regions such as the thalamus, hypothalamus, and hippocampus (6). In contrast, GAD is mainly characterized by dysfunction in emotional regulation networks, particularly involving the prefrontal cortex, amygdala, and insula (7). In cases of comorbidity, abnormalities may coexist in both sleep and emotional regulation systems. These differences suggest that CID, GAD, and their comorbidity may exhibit distinct neuroanatomical features. These features could be useful for differential diagnosis.

Our previous study reported preliminary findings of patients with CID comorbid GAD (8). However, that study was limited by a relatively simple design, as it did not include independent CID and GAD groups for comparison. To date, relatively few studies have systematically investigated the neuroanatomical features of comorbidity. Most existing research has focused on CID or GAD separately. Prior studies have identified neuroanatomical abnormalities in the prefrontal cortex, thalamus, and hippocampus in patients with CID (9). Similarly, neuroanatomical alterations in the amygdala, prefrontal cortex, and insula have been reported in patients with GAD (10). Among available neuroanatomical techniques, magnetic resonance imaging (MRI)-based measurement of gray matter offers several advantages. It is a rapid and routinely used examination, and gray matter morphology remains relatively stable over extended periods. Therefore, MRI-based gray matter measurement may represent a promising tool for improving the objective diagnosis of CID, GAD, and their comorbidity.

In this study, we aimed to identify neuroanatomical features of CID, GAD, and CID comorbid GAD. We also explored their potential utility in disease classification. Compared with previous studies, our study has several strengths. Firstly, we included four well-defined groups: CID, GAD, CID comorbid GAD, and healthy controls. Secondly, we evaluated multiple gray matter metrics, including fractal dimension (FD), gyrification index (GI), and regional gray matter volume proportion. These metrics are objective and relatively robust to individual variability. In summary, we hope that neuroanatomical features can aid diagnosis in clinical practice. This may enable accurate identification of CID, GAD, and CID comorbid GAD in the future.

## 2 Materials and methods

### 2.1 Participant screening

Participant screening was conducted by two senior psychiatrists with extensive clinical experience. The illness duration and clinical condition of all participants were fully recorded. Diagnosis was based on the Diagnostic and Statistical Manual of Mental Disorders Fifth Edition (DSM-5) and the International Classification of Sleep Disorders Third Edition (ICSD-3). These manuals provide standardized diagnostic criteria for CID and GAD. Two psychiatrists independently made diagnoses in a blinded manner. They were instructed to pay particular attention to distinguishing depression and other psychiatric disorders. Only participants with consistent diagnoses from both psychiatrists were included in the study. In addition, all participants were required to complete questionnaires for clinical assessment. CID severity was assessed using the Insomnia Severity Index (ISI) and the Pittsburgh Sleep Quality Index (PSQI). GAD severity was evaluated using the Hamilton Anxiety Rating Scale (HAMA) and the Self-Rating Anxiety Scale (SAS). Somatic symptom was assessed using the Somatic Symptom Scale (SSS). Cognitive function was assessed using the Montreal Cognitive Assessment (MoCA). Participants were included in the study only when their questionnaire results were consistent with the clinical diagnoses. Subsequently, two psychiatrists carefully assessed the medication history of all participants. All participants reported their medication history during the previous year. These records were documented and reviewed. Only participants who had not taken psychiatric medications within the previous two weeks were included in the study.

### 2.2 Inclusion and exclusion criteria

A total of 46 patients with CID, 44 patients with GAD, 43 patients with CID comorbid GAD, and 45 healthy controls (HC) were included in this study.

For the CID group, the inclusion criteria were: (a) age 18–55 years, Han Chinese ethnicity, and right-handedness; (b) diagnosis of CID according to the ICSD-3; (c) diagnosis of insomnia according to the DSM-5; (d) PSQI score > 7 and ISI score > 15; (e) no other psychiatric or sleep disorders; (f) no use of psychotropic medications within 2 weeks before the study.

For the GAD group, the inclusion criteria were: (a) age 18–55 years, Han Chinese ethnicity, and right-handedness; (b) diagnosis of GAD according to the DSM-5; (c) SAS score > 50 and HAMA score > 14; (d) no other psychiatric or sleep disorders; (e) no use of psychotropic medications within 2 weeks before the study.

For the CID comorbid GAD group, the inclusion criteria were: (a) age 18–55 years, Han Chinese ethnicity, and right-handedness; (b) diagnosis of GAD according to the DSM-5; (c) diagnosis of CID according to the ICSD-3; (d) SAS score > 50 and HAMA score > 14; (e) PSQI score > 7 and ISI score > 15; (f) no other psychiatric or sleep disorders; (7) no use of psychotropic medications within 2 weeks before the study.

For the HC group, the inclusion criteria were: (a) age 18–55 years, Han Chinese ethnicity, and right-handedness; (b) good emotional status and sleep quality, with normal cognitive function; (c) no other psychiatric or sleep disorders.

All groups followed the exclusion criteria: (a) history of head trauma or structural brain abnormalities on routine MRI; (b) pregnancy, lactation, or menstruation; (c) smoking, alcohol abuse, illicit drug use, tea drinking, or coffee consumption within 24 hours before the study; (d) MRI contraindications, including metallic implants or claustrophobia.

### 2.3 MRI data acquisition

All participants were scanned using a 3.0 Tesla MRI scanner (Siemens, Germany) with a 64-channel head coil. The whole-brain structural images were acquired using a sagittal 3D-magnetization-prepared rapid acquisition gradient echo (MPRAGE) sequence. The sequence with the following parameters: Repetition time (TR)= 2300 ms; Echo time (TE)= 2.98 ms; Inversion time (TI)= 900 ms; Flip angle (FA)= 9°; Field of view (FOV)= 256 x 256 mm^2^; Slice thickness= 1 mm; voxel size=1.0 x 1.0 x 1.0 mm. In addition, an axial T2-weighted fluid-attenuated inversion recovery (T2-FLAIR) sequence was acquired to exclude organic brain lesions. Participants with structural abnormalities or motion artifacts on the images were excluded from the study. Two experienced radiologists were invited to evaluate the MRI scans.

### 2.4 VBM analysis

In this study, VBM analysis was used to obtain gray matter volume (GMV) for each brain region in all participants. The VBM analysis was performed as follows: (a) MATLAB software was launched and the SPM12 and CAT12 toolboxes were added. (b) Convert DICOM files to NIFITI images; (c) Screen for artifacts or anatomical abnormalities; (d) Manually re-oriented to the anterior; (e) Spatial registration of T1-weighted images to a reference brain template; (f) Segmentation of images into gray matter, white matter, and cerebrospinal fluid; (g) Perform the DARTEL method for registration, normalization, and modulation; (h) The Jacobian determinants derived from spatial normalization that modulate the image intensity of each voxel were used to verify regional differences in the conservation of the total amount of gray matter; (i) Transform images to standard MNI space; (j) Spatial Smoothing using a Gaussian kernel with a 8 mm FWHM; (k) The AAL atlas and hippocampal subregion atlas were used to parcellate the brain; (l) The GMV for each brain region were obtained and visualized. Subsequently, analysis of ANCOVA was performed using the DPABI toolbox to compare regional GMV among the four groups. The *Post hoc* two-sample t tests were further conducted. Age, sex, illness duration and years of education were included as covariates in all analyses, and p < 0.05 was considered statistically significant. The two-tailed GRF correction was used for multiple comparisons, with a voxel-level threshold of p < 0.001 and a cluster-level threshold of p < 0.05. Finally, GMV values of the significantly altered brain regions were extracted and visualized.

### 2.5 SBM analysis

In this study, SBM analysis was used to obtain fractal dimension (FD) and gyrification index (GI) for each brain region in all participants. The SBM analysis was performed as follows: (a) Image segmentation, cortical reconstruction, topological correction, spherical registration, and spatial normalization were used the same steps above; (b) Central surface images of the left and right cerebral hemispheres were generated using CAT12 toolbox and DARTEL algorithm; (c) The resulting images were subjected to quality control; (d) The Surface Tools module in CAT12 was used to extract the GI and FD from the central surfaces of both hemispheres; (e) GI and FD maps were smoothed using a 20 mm FWHM Gaussian kernel; (f) The DKT40 atlas was used to parcellate the brain; (g) GI and FD values were extracted and visualized. Subsequently, analysis of ANCOVA was performed using the CAT12 and SPM12 toolboxes to compare regional GI and FD among the four groups. The *Post hoc* two-sample t tests with Bonferroni correction were further conducted. Age, sex, illness duration and years of education were included as covariates in all analyses, and p < 0.05 was considered statistically significant. The FWE correction was used for multiple comparisons, with a significance threshold of p < 0.05. Finally, GI and FD values of the significantly altered brain regions were extracted and visualized.

### 2.6 Regional volume fraction analysis

The regional volume fraction was derived from VBM analysis. It is a more objective and practical measure than regional GMV alone. As a proportional measure, it reduces the influence of individual differences in brain size. The procedure was performed as follows: (a) The whole-brain volume was calculated for each participant; (b) The brain was parcellated into different regions using brain atlases, and the GMV of each region was extracted; (c) The regional GMV was divided by the whole-brain volume to obtain the regional volume fraction.

### 2.7 Statistical analysis

Statistical analysis was performed using SPSS 22.0 software. Prior to analysis, data were examined for outliers, skewness, and homogeneity of variance to ensure the appropriateness of statistical tests. ANCOVA and t-test were used to analyze group differences in demographic variables and survey scores among the four experimental groups. Chi-square tests were used to analyze gender differences between groups. A significance level of p < 0.05 was considered statistically significant.

Partial correlation analysis was conducted separately in each group to examine the relationship between brain region parameters and clinical characteristics. The brain region parameters included GMV, FD, and GI values. The clinical characteristics included HAMA scores, SAS scores, ISI scores, PSQI scores, and MoCA scores. Gender, age, illness duration and years of education were included as covariates in the analysis. A significance level of p < 0.05 was considered statistically significant. To control for multiple comparisons, the false discovery rate (FDR) correction was applied to the p values obtained from the correlation analyses within each group. Statistical significance was defined as FDR-corrected p < 0.05.

### 2.8 Ethics statement

This study was approved by the Ethics Committee of the Shengjing Hospital of China Medical University (approval number 2025PS1399K). It was conducted in accordance with the principles of the Declaration of Helsinki. All participants were recruited through hospital and community advertisements. They voluntarily participated in the study and received both written and oral explanations regarding study. Written informed consent was obtained from all participants.

## 3 Results

### 3.1 Participant demographics

The four groups (CID group, GAD group, CID comorbid GAD group, HC group) were compared. The results showed no significant differences in age, gender, illness duration, education years, body mass index, and cognitive function. In contrast, significant differences were observed in anxiety, insomnia, and somatization scale scores (**Table 1**).

**Table 1:** Participant demographics.

Characteristics	CID (n=46)	GAD (n=44)	CID comorbid GAD (n=43)	HC (n=45)	F/X2	*P* value
Age (years old)	38.67 ± 10.66	38.66 ± 11.70	38.37 ± 10.51	39.11 ± 10.90	0.03^a^	0.99
Gender (M/F)	25/21	23/21	23/20	22/23	0.31^b^	0.96
illness duration (months)	3.26 ± 0.29	3.24 ± 0.27	3.16 ± 0.24	—	1.60^a^	0.21
Education (years)	11.83 ± 4.48	11.57 ± 4.40	11.72 ± 4.48	11.67 ± 4.40	0.03^a^	0.99
body mass index	20.94 ± 1.20	21.06 ± 1.10	21.06 ± 0.96	20.61 ± 1.02	1.76 ^a^	0.16
MoCA scores	28.98 ± 0.83	28.98 ± 0.82	28.98 ± 0.83	29.00 ± 0.83	0.01^a^	1.00
HAMA scores	3.30 ± 1.71	30.64 ± 4.17	41.16 ± 2.91	2.00 ± 1.07	2322.81^a^	0.00**
SAS scores	11.72 ± 6.39	62.64 ± 4.14	58.33 ± 3.75	2.93 ± 1.30	2287.94^a^	0.00**
ISI scores	20.89 ± 1.95	2.20 ± 1.03	20.56 ± 1.92	2.27 ± 1.03	2117.31^a^	0.00**
PSQI scores	15.41 ± 1.68	2.50 ± 1.05	15.16 ± 1.63	1.40 ± 0.50	1554.91^a^	0.00**
SSS scores	35.11 ± 4.45	35.14 ± 4.20	33.16 ± 4.60	2.67 ± 1.38	755.22^a^	0.00**

The comparison results of demographic characteristics and questionnaire scores between groups. Demographic characteristics included age, gender, illness duration, education years, and body mass index. Questionnaire scores include MoCA, HAMA, SAS, ISI, PSQI, and SSS scores. The comparison was performed using ANCOVA test and *X*^2^ test. ^a^ANCOVA test, ^b^*X*^2^ test, ^*^p < 0.05, ^**^p < 0.01. Values are reported as mean and standard deviation, except for gender. CID, chronic insomnia disorder; GAD, generalized anxiety disorder; HC, healthy control; M, male; F, female; MoCA, Montreal Cognitive Assessment Scale; HAMA, Hamilton Anxiety Scale; SAS, Self-rating Anxiety Scale; ISI, Insomnia Severity Index Scale; PSQI, Pittsburgh Sleep Quality Index; SSS, Somatic Symptom Scale.

### 3.2 VBM comparison results

**Figure 1:**
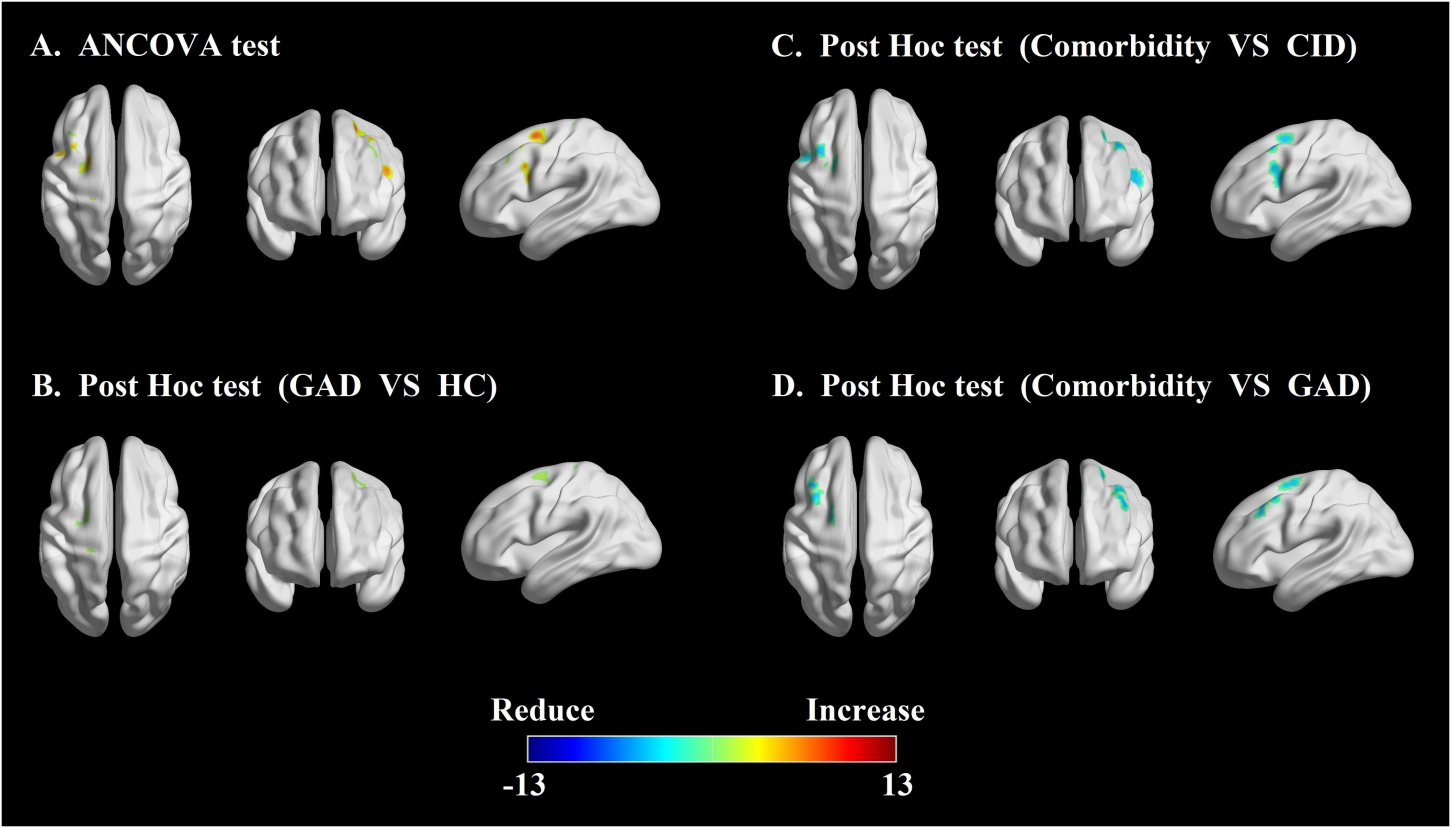
VBM results of the ANOVA and *Post Hoc* tests. **(A)** ANOVA results. Significant GMV differences in the left middle frontal gyrus. **(B)** *Post Hoc* results for the GAD and HC groups comparison. Significant GMV increased in the left precentral gyrus. **(C)** *Post Hoc* results for the Comorbidity and CID groups comparison. Significant GMV reduced in the left middle frontal gyrus. **(D)** *Post Hoc* results for the Comorbidity and GAD groups comparison. Significant GMV reduced in the left middle frontal gyrus.

**Figure 2:**
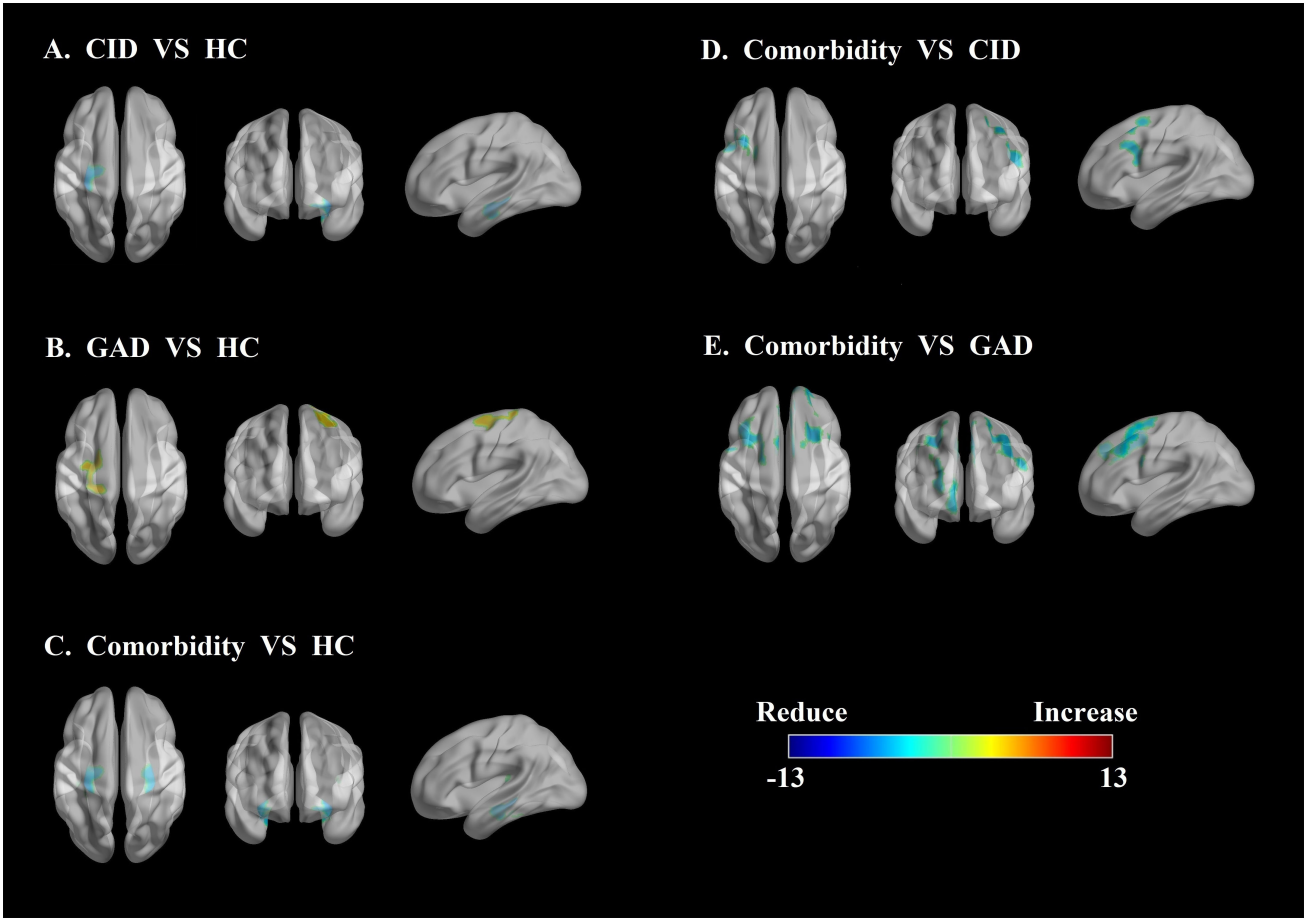
VBM results of the two-sample-t-tests. **(A)** Comparison results between the CID and HC groups. Significant GMV reduced in the left hippocampus. **(B)** Comparison results between the GAD and HC groups. Significant GMV increased in the left precentral gyrus. **(C)** Comparison results between the Comorbidity and HC groups. Significant GMV reduced in the bilateral hippocampus. **(D)** Comparison results between the Comorbidity and CID groups. Significant GMV reduced in the left middle frontal gyrus. **(E)** Comparison results between the Comorbidity and GAD groups. Significant GMV reduced in the right middle frontal gyrus and the right superior frontal gyrus.

The ANCOVA revealed significant GMV differences in a large region of the left prefrontal cortex (**Figure 1**). Two-sample-t-tests was conducted to compare the differences between HC group and patient groups (**Figure 2**). The CID group showed a reduction in the left hippocampus. The GAD group showed an increase in the left precentral gyrus. The CID comorbid GAD group showed a reduction in the bilateral hippocampus. Additionally, comparisons were conducted between the comorbid group and either the CID group or the GAD group. The CID comorbid GAD group showed a reduction in the prefrontal cortex. These findings were further supported by the *post hoc* tests. (**Table 2**).

**Table 2:** VBM Comparison results.

Comparison groups	Trend	Cluster peak	Cluster size	MNl coordinates			T/F value	*P* value
				X	Y	Z		
CID VS HC^a^	reduce	Hippocampus_L	970	-34.5	-25.5	-4.5	-6.36	0.00**
GAD VS HC^a^	increase	Precentral_L	740	-27	-19.5	60	5.36	0.00**
comorbidity VS HC^a^	reduce	Hippocampus_L	1078	-30	-28.5	-9	-5.95	0.00**
	reduce	Hippocampus_R	932	28.5	-16.5	-19.5	-5.16	0.00**
comorbidity VS CID^a^	reduce	Frontal_Mid_L	936	-36	13.5	51	-5.08	0.00**
comorbidity VS GAD^a^	reduce	Frontal_Mid_R	906	27	22.5	52.5	-5.44	0.00**
	reduce	Frontal_Sup_R	448	16.5	48	12	-4.82	0.00**
ANCOVA^b^	Difference	Frontal_Mid_L	1394	-34.5	10.5	45	12.44	0.00**
GAD VS HC^c^	increase	Precentral_L	494	-27	-19.5	60	5.36	0.00**
comorbidity VS CID^c^	reduce	Frontal_Mid_L	673	-36	13.5	51	-5.08	0.00**
comorbidity VS GAD^c^	reduce	Frontal_Mid_L	705	-1.5	13.5	46.5	-5.21	0.00**

The comparison results of VBM analysis between groups. The comparison was performed using T-test, ANCOVA test, and *post hoc* test. ^a^T-test, ^b^ANCOVA test, ^c^*post hoc* test, ^*^p < 0.05, ^**^p < 0.01. VBM, Voxel-based morphometry; CID, chronic insomnia disorder group; GAD, generalized anxiety disorder group; HC, healthy control group; MNI, Montreal Neurological Institute;L, left; R, right.

### 3.3 SBM comparison results

**Figure 3:**
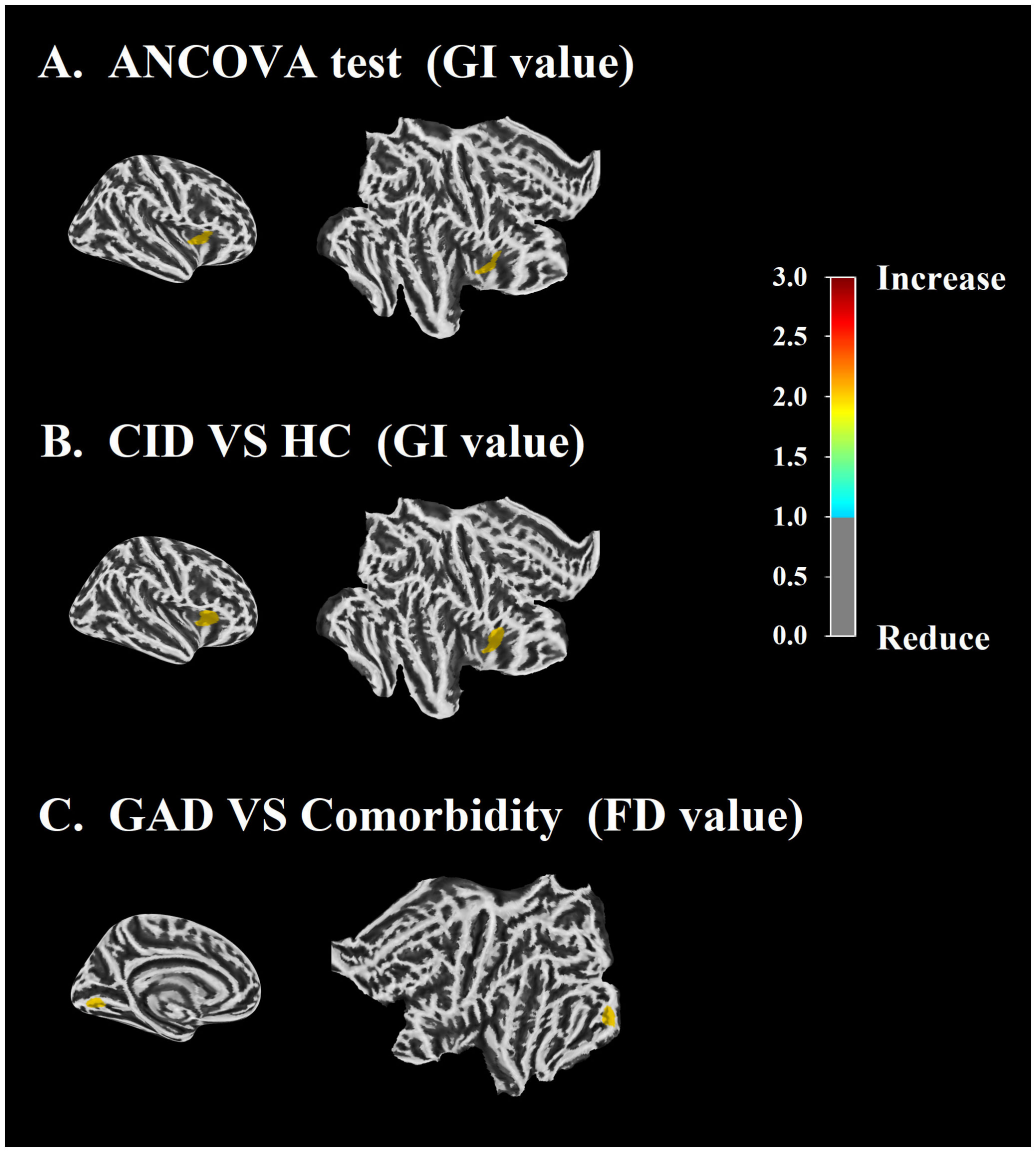
SBM results of the ANOVA and two-sample-t-tests. **(A)** ANOVA of GI value. Significant GI differences in the right insula, right pars opercularis, and right pars triangularis. **(B)** GI value comparison between the CID and HC groups. Significant GI increased in the right pars triangularis, right pars opercularis, and right insula. **(C)** FD value comparison between the GAD and Comorbidity groups. Significant FD increased in the left lingual gyrus.

The ANCOVA revealed significant GI differences in the right insula, right pars opercularis, and right pars triangularis. Two-sample-t-tests was conducted to compare the differences between HC group and patient groups. The CID group showed increased GI values in the right pars triangularis, right pars opercularis, and right insula. Additionally, the GAD group showed an increased FD in the left lingual than the comorbidity group. (**Table 3**; **Figure 3**).

**Table 3:** SBM comparison results.

Comparison groups	Index	Trend	Cluster size	Overlap of atlas region	T/F value	*P* value
CID VS HC^a^	GI value	increase	207	35% right pars triangularis	2	0.03*
				34% right pars opercularis	2	0.03*
				30% right insula	2	0.03*
GAD VS comorbidity^a^	FD value	increase	120	100% left lingual	2	0.03*
ANCOVA^b^	GI value	Difference	126	68% right insula	2	0.03*
				21% right pars opercularis	2	0.03*
				11% right pars triangularis	2	0.03*

The comparison results of SBM analysis between groups. The comparison was performed using T-test and ANCOVA test. ^a^T-test, ^b^ANCOVA test, ^*^p < 0.05, ^**^p < 0.01. SBM, Surface-based morphometry; CID, chronic insomnia disorder group; GAD, generalized anxiety disorder group; HC, healthy control group; GI, gyrification index;FD, fractal dimension.

### 3.4 Regional volume fraction comparison results

**Figure 4:**
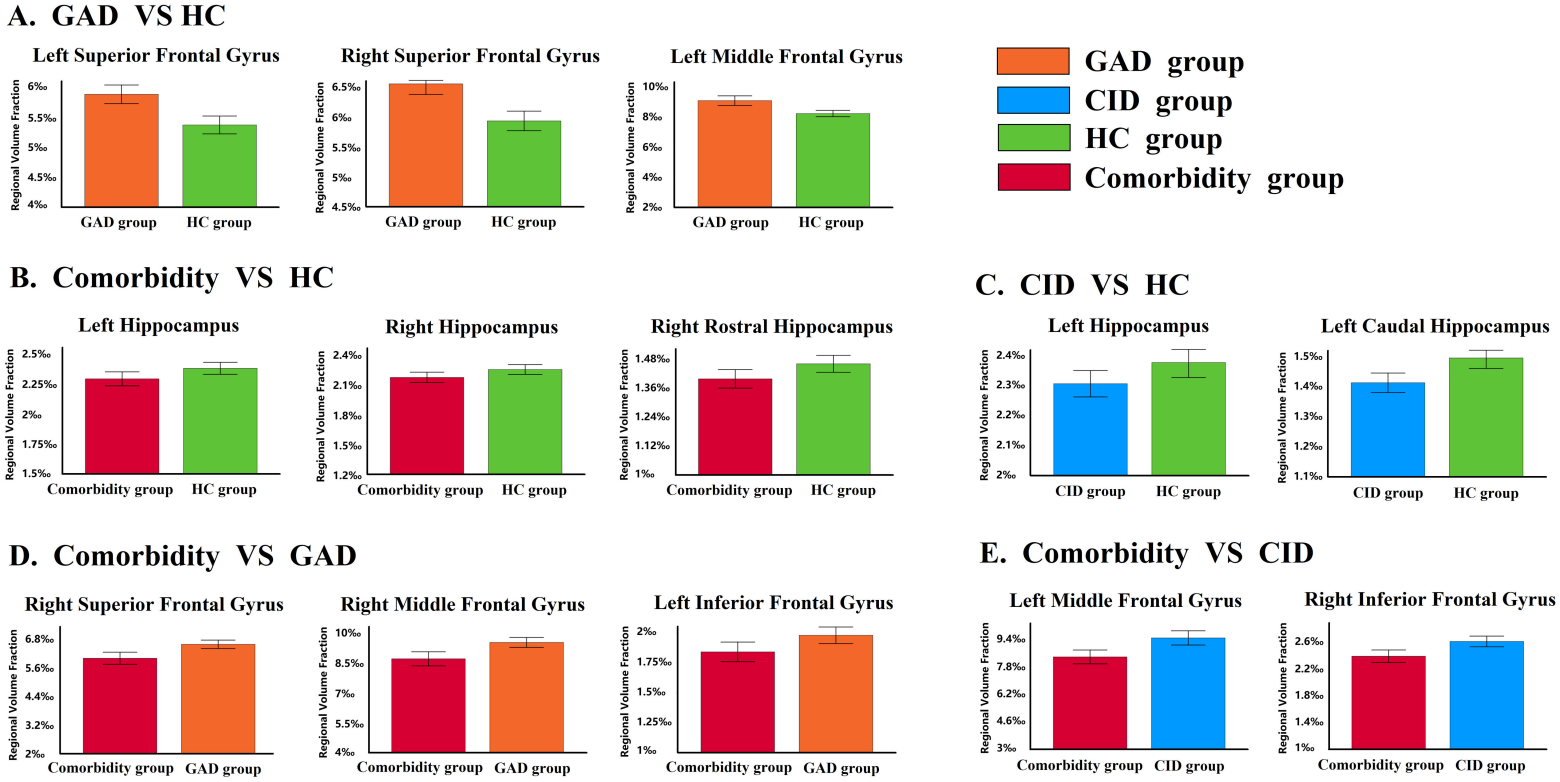
Comparison results for regional volume fraction values. **(A)** Bar chart of the comparison results between the GAD and HC groups. **(B)** Bar chart of the comparison results between the Comorbidity and HC groups. **(C)** Bar chart of the comparison results between the CID and HC groups. **(D)** Bar chart of the comparison results between the Comorbidity and GAD groups. **(E)** Bar chart of the comparison results between the Comorbidity and CID groups. Errors bar showed standard error.

ANCOVA and t-test were used to compare intergroup differences in regional volume fraction. The ANCOVA revealed significant differences in the prefrontal cortex and hippocampus. The t-test revealed that the left hippocampus was smaller in the CID group than in the HC group. The prefrontal cortex was larger in the GAD group than in the HC group. The bilateral hippocampus was smaller in the comorbidity group than in the HC group. The prefrontal cortex was smaller in the comorbidity group than in the CID group. The prefrontal cortex was smaller in the comorbidity group than in the GAD group. (**Table 4**; **Figure 4**).

**Table 4:** Regional volume fraction comparison results.

Comparison groups	Brain regions	Trend	T/F value	*P* value
CID VS HC^a^	Hippocampus_L	reduce	-2.12	0.04*
	L_caudal hippocampus	reduce	-3.47	0.00**
GAD VS HC^a^	Frontal_Sup_L	increase	4.75	0.00**
	Frontal_Sup_R	increase	5.15	0.00**
	Frontal_Mid_L	increase	4.50	0.00**
comorbidity VS HC^a^	Hippocampus_L	reduce	-2.29	0.03*
	Hippocampus_R	reduce	-2.18	0.03*
	R_rostral hippocampus	reduce	-2.39	0.02*
comorbidity VS CID^a^	Frontal_Mid_L	reduce	-3.83	0.00**
	Frontal_Inf_Oper_R	reduce	-3.61	0.00**
comorbidity VS GAD^a^	Frontal_Sup_R	reduce	-3.78	0.00**
	Frontal_Mid_R	reduce	-3.82	0.00**
	Frontal_Inf_Oper_L	reduce	-2.59	0.01*
ANCOVA^b^	Frontal_Sup_L	Difference	8.57	0.00**
	Frontal_Sup_R	Difference	11.48	0.00**
	Frontal_Mid_L	Difference	11.54	0.00**
	Frontal_Mid_R	Difference	11.14	0.00**
	Frontal_Inf_Oper_L	Difference	11.37	0.00**
	Frontal_Inf_Oper_R	Difference	5.92	0.00**
	Hippocampus_L	Difference	2.82	0.04*
	L_caudal hippocampus	Difference	4.37	0.01*
	R_rostral hippocampus	Difference	4.91	0.00**

The comparison results of Regional Volume Fraction between groups. The comparison was performed using T-test and ANCOVA test. ^a^T-test, ^b^ANCOVA test, ^*^p < 0.05, ^**^p < 0.01. CID, chronic insomnia disorder group; GAD, generalized anxiety disorder group; HC, healthy control group; L, left; R, right.

### 3.5 Correlation analysis results

**Figure 5:**
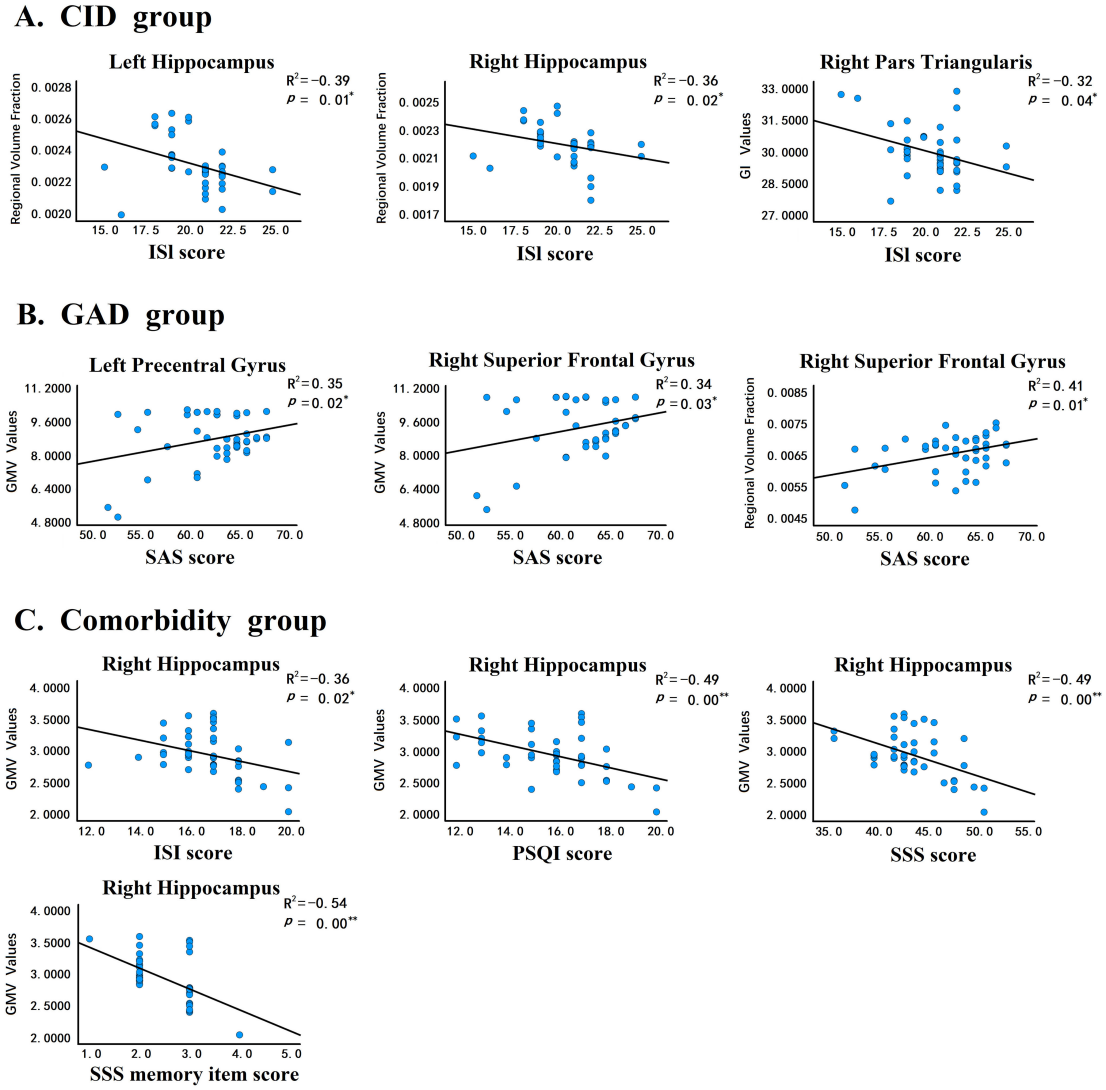
The significant results of partial correlation analysis between brain parameters and scale scores. FDR correction has been performed. **(A)** Scatter plot of partial correlations in the CID group. **(B)** Scatter plot of partial correlations in the GAD group. **(C)** Scatter plot of partial correlations in the Comorbidity group. ^*^p < 0.05, ^**^p < 0.01.

Partial correlation analysis revealed the relationship between brain parameters and scale scores. The FDR correction has been performed. In the CID group, the ISI score was negatively correlated with the regional volume fraction of the bilateral hippocampus. The ISI score was also negatively correlated with the GI value of the right pars triangularis. In the GAD group, the SAS score was positively correlated with the GMV and the regional volume fraction of the right superior frontal gyrus. The SAS score was also positively correlated with the GMV of the left precentral gyrus. In the comorbidity group, the GMV of the right hippocampus was negatively correlated with the ISI score, PSQI score, SSS score, SSS memory item score (**Table 5**; **Figure 5**).

**Table 5:** Partial correlation analysis results.

Groups	Brain parameters	Brain regions	Scale scores	r value	*P* value	FDR-corrected *P* value
CID	Regional Volume Fraction	Hippocampus_L	ISI score	-0.39	0.01*	0.03*
	Regional Volume Fraction	Hippocampus_R	ISI score	-0.36	0.02*	0.03*
	GI value	right pars triangularis	ISI score	-0.32	0.04*	0.04*
GAD	GMV value	Precentral_L	SAS score	0.35	0.02*	0.03*
	GMV value	Frontal_Sup_R	SAS score	0.34	0.03*	0.03*
	Regional Volume Fraction	Frontal_Sup_R	SAS score	0.41	0.01*	0.03*
Comorbidity	GMV value	Hippocampus_R	ISI score	-0.36	0.02*	0.02*
			PSQI score	-0.49	0.00**	0.01*
			SSS score	-0.49	0.00**	0.01*
			SSS memory item score	-0.54	0.00**	0.01*

The significant results of partial correlation analysis between brain parameters and scale scores. Brain parameters included Regional Volume Fraction, GI value, and GMV value. Scale scores include ISI, SAS, PSQI, SSS, and SSS memory item score. CID, chronic insomnia disorder group; GAD, generalized anxiety disorder group; GMV, gray matter volume; GI, gyrification index; ISI, Insomnia Severity Index Scale; SAS, Self-rating Anxiety Scale; PSQI, Pittsburgh Sleep Quality Index; SSS, Somatic Symptom Scale; FDR, False Discovery Rate; L, left; R, right; ^*^p < 0.05, ^**^p < 0.01.

## 4 Discussion

Three years ago, our team conducted a study on patients with CID comorbid GAD (8). We found atrophy in certain brain regions that was associated with the disease. In subsequent research, we aimed to include additional experimental groups for a more detailed investigation. In this study, we recruited patients with three different disease states: pure CID, pure GAD, and CID comorbid GAD. Using VBM analysis, we identified structural differences in the brains of these three patient groups. Specifically, differences were observed in the prefrontal cortex and hippocampus. Moreover, these differences were significantly associated with insomnia and anxiety. Therefore, we suggest that abnormalities in the prefrontal cortex and hippocampus may serve as neuroanatomical feature for clinical diagnosis. Next, we will elaborate on this conclusion.

### 4.1 Neuroanatomical feature of CID

In the VBM analysis, we found that the left hippocampus was significantly reduced in patients with CID. Partial correlation analysis further showed that hippocampal volume was negatively correlated with insomnia severity. In the SBM analysis, patients with CID showed a significantly increased GI in the prefrontal cortex, and this alteration was also negatively correlated with insomnia severity. We believe that both abnormalities may represent neuroanatomical features of CID. From a physiological perspective, the hippocampus is closely related to sleep. It is generally believed that the hippocampus can maintain normal structure and function only under conditions of good sleep (11). Insomnia may lead to hippocampal atrophy (12, 13). In addition, our study also found an abnormally increased GI in the prefrontal cortex of patients with CID. This finding may be explained by the cortical tension theory (14). According to this theory, long-term neural hyperexcitability in insomnia may increase the density of cortical neural connections. This may further enhance axonal tension (15). As a result, cortical folding may become more complex (16), leading to an increased GI. It is also worth noting that our findings have been mentioned in previous studies. In recent years, some researchers have investigated both psychophysiological insomnia and paradoxical insomnia. They found that both types of insomnia were associated with reduced hippocampal gray matter volume (17). In addition, another research team reviewed related studies published over the past two decades. They reported that hippocampal volume reduction was documented in most studies on insomnia (18, 19). On the other hand, previous SBM studies have also shown that insomnia may lead to an increased GI in the prefrontal cortex (20). Taken together, we believe that hippocampal reduction may serve as a neuroanatomical feature of CID and may help improve clinical diagnosis.

### 4.2 Neuroanatomical feature of GAD

In this study, we found increased gray matter volume in the left precentral gyrus of patients with GAD. Partial correlation analysis further showed that this alteration was positively correlated with anxiety severity. The regional volume fraction was also increased in the left superior frontal gyrus, right superior frontal gyrus, and left middle frontal gyrus. Although the differences in prefrontal regions may be due to the stringent correction used in the VBM analysis. However, these findings suggest that the prefrontal cortex is a region worthy of further study in GAD patients. In contrast, patients with CID and comorbidity showed gray matter atrophy. This finding was clearly different from the increased GMV observed in patients with GAD. Therefore, we believe that prefrontal enlargement may represent a neuroanatomical feature of GAD. It may also be useful for the differential diagnosis and clinical identification of GAD. In fact, many previous studies have also reported abnormal prefrontal changes in anxiety. One research group used machine learning methods to predict anxiety. They found that the prediction accuracy exceeded 75% when prefrontal volume was used as a feature (21). In another large scale study with 12,000 participants, the results also showed a strong association between prefrontal abnormalities and anxiety (22). Studies in infants have provided further evidence. Researchers found that the prefrontal cortex could even predict emotional outcomes in infants (23). Specifically, a larger prefrontal cortex was associated with a higher likelihood of later negative emotions. If the prefrontal cortex was relatively large at 3 months of age, the infant was more likely to show negative emotions in the following period. In addition, new findings have continued to emerge with more researches. In a recent study on the gut and brain, researchers suggested that prefrontal enlargement in anxiety may be related to the gut microbiota (24). In studies of cognitive behavior, researchers found that abnormal enlargement of the prefrontal cortex was associated with impaired fear extinction (25) and attentional dysfunction (26). Some physiologists also support this finding (27, 28). The prefrontal alteration may serve as a neuroanatomical feature to assist in the diagnosis of GAD.

### 4.3 Neuroanatomical feature of CID comorbid GAD

In this study, we found that the comorbidity group showed significant differences from the other groups. Specifically, both the hippocampus and prefrontal cortex were significantly reduced in the comorbidity group. The extent and severity of brain atrophy were also greater than those observed in patients with CID alone. We therefore believe that widespread atrophy of the hippocampus and prefrontal cortex may represent a neuroanatomical feature of CID comorbid GAD. From a physiological perspective, this finding may result from the combined effects of CID and GAD. These two conditions share several common mechanisms (29). In addition, CID comorbid with GAD reflects abnormalities in both sleep regulation and emotion regulation (30, 31). Therefore, the prefrontal cortex, which is involved in emotional regulation, would also be expected to show abnormal changes (32). This pattern is different from that seen in CID alone. The damaging effect of comorbidity on brain atrophy appears to be much greater than that of CID alone. We therefore believe that the main difference between comorbidity and pure CID is that comorbidity leads to more widespread brain atrophy. This may be the key point for distinguishing comorbidity from pure CID. If both the prefrontal cortex and hippocampus show atrophy, this may suggest the presence of CID comorbid with GAD. In contrast, the characteristic feature of pure GAD is abnormal enlargement of the prefrontal cortex. This forms a clear contrast with the gray matter reduction observed in CID comorbid with GAD (33). However, when anxiety is accompanied by insomnia, brain atrophy may become the dominant pattern. From a physiological perspective, this may indicate that the damaging effect of CID is greater than that of GAD (34). The CID keeps brain in an excited state during both the daytime and nighttime (35). This deprives GAD related brain dysfunction of its only opportunity for repair during the night (36). Therefore, when GAD is accompanied by CID, the destructive effect of CID may become dominant and lead to more extensive brain atrophy (37). In our partial correlation analysis, we found that the degree of hippocampal reduction was negatively correlated with insomnia severity. This further supports the contribution of CID to brain atrophy. We also found that hippocampal volume was negatively correlated with memory scale scores. This suggests that hippocampal shrinkage is not only a morphological alteration, but also impairs memory function (38). Taken together, we believe that reduced GMV in the prefrontal cortex and hippocampus may represent a neuroanatomical feature of CID comorbid with GAD.

### 4.4 Limitations and prospects

In this study, we aimed to identify neuroanatomical features of CID, GAD, and their comorbidity. We also hoped to distinguish these conditions from each other. This remains a major challenge in current clinical practice (39). Our findings suggest that abnormalities in the prefrontal cortex and hippocampus may help differentiate these conditions. To make the results more objective, we also included regional brain volume fractions and SBM analysis. However, our study also has several limitations. Firstly, the study is mainly based on group comparisons and partial correlations. No formal classification model, cross-validation, or AUC analysis is presented. Therefore, we hope that our study may provide some guidance and inspiration for future researchers. Secondly, the relatively small sample size may have affected the reliability of the results. We did not perform a longitudinal study to track disease progression. Thirdly, we did not carefully distinguish between different disease subtypes. We acknowledge that this is only a preliminary study. We simply hope that it may provide some inspiration for future research. In the next step, we plan to apply current machine learning (40, 41) and deep learning (42, 43) methods to improve the accuracy of classification. We hope to build efficient and reliable classification models.

## Data Availability

The raw data supporting the conclusions of this article will be made available by the authors, without undue reservation.
